# Online clinical tools to support the use of new plasma biomarker diagnostic technology in the assessment of Alzheimer’s disease: a narrative review

**DOI:** 10.1093/braincomms/fcad322

**Published:** 2023-11-24

**Authors:** Jemma Hazan, Kathy Y Liu, Nick C Fox, Robert Howard

**Affiliations:** Division of Psychiatry, University College London, London W1T 7BN, UK; Division of Psychiatry, University College London, London W1T 7BN, UK; Dementia Research Centre, UCL Queen Square Institute of Neurology, University College London, London, WC1N 3BG, UK; UK Dementia Research Institute at UCL, London, W1T 7NF, UK; Division of Psychiatry, University College London, London W1T 7BN, UK

**Keywords:** Alzheimer’s disease, dementia, biomarker, diagnosis, investigation

## Abstract

Recent advances in new diagnostic technologies for Alzheimer’s disease have improved the speed and precision of diagnosis. However, accessing the potential benefits of this technology poses challenges for clinicians, such as deciding whether it is clinically appropriate to order a diagnostic test, which specific test or tests to order and how to interpret test results and communicate these to the patient and their caregiver. Tools to support decision-making could provide additional structure and information to the clinical assessment process. These tools could be accessed online, and such ‘e-tools’ can provide an interactive interface to support patients and clinicians in the use of new diagnostic technologies for Alzheimer’s disease. We performed a narrative review of the literature to synthesize information available on this research topic. Relevant studies that provide an understanding of how these online tools could be used to optimize the clinical utility of diagnostic technology were identified. Based on these, we discuss the ways in which e-tools have been used to assist in the diagnosis of Alzheimer’s disease and propose recommendations for future research to aid further development.

## Teaser

Hazan *et al*. summarize the ways in which online clinical tools could be used to support clinicians and patients, in a memory clinic setting, in the diagnosis of Alzheimer’s disease. This is increasingly important, specifically for clinics that implement plasma biomarkers. Further work is required to design and integrate related tools.

## Graphical Abstract

**Graphical Abstract:**
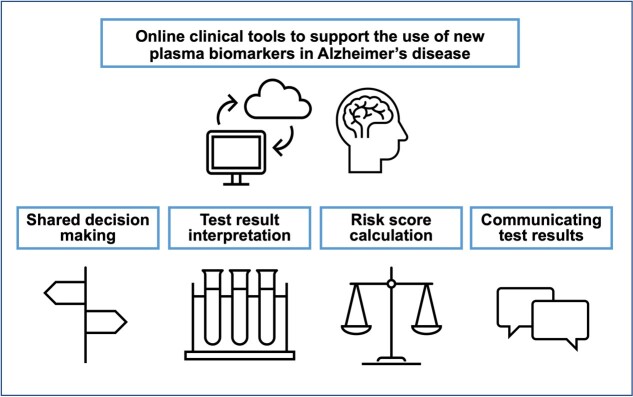

## The evolution of diagnostic technology for Alzheimer’s disease

Diagnostic technologies for Alzheimer’s disease have seen large advances in the last decade, with extensive research in the discovery and validation of new biomarker technology. There is an increasing focus on their use in both the research and clinical domains, to increase certainty in diagnosis, particularly with the advent of potential disease-modifying treatments. Such biomarkers can be used to detect *in vivo* pathological hallmarks of Alzheimer’s disease. A diagnostic research framework was proposed in 2018 with a shift in the definition of Alzheimer’s disease to a biological construct.^1^ Within this, biomarkers are grouped into three categories to reflect amyloid deposition, tau pathology, and neurodegeneration. The recommendations set out in the 2021 International Working Group by Dubois *et al.* in *Lancet Neurology* provide guidance on incorporating Alzheimer’s disease biomarkers to aid in the diagnosis of Alzheimer’s disease in clinical practice.^2,3^ The National Institute on Aging and the Alzheimer’s Association’s Clinical Guidelines for Alzheimer’s Disease were revised in 2023. They recommend that a diagnosis of Alzheimer’s disease should be defined biologically and through *in vivo* detection of abnormal biomarkers.^4^

Neuroimaging using MRI or CT of the brain can provide information on the effects of disease, such as patterns of atrophy and hippocampal volume loss.^5^ Newer neuroimaging techniques such as amyloid-PET provide the evidence of amyloid deposition in the brain.^6^ Such diagnostic technology may reveal disease pathology, which may or may not be clinically apparent at the time of scan. Moreover, such scans are relatively costly and scarce, and require intravenous radioactive tracers.^7-9^ Nevertheless, studies have shown that amyloid-PET has clinical utility in US community settings with implications for the number of Alzheimer’s disease diagnoses made and patient management.^9^

Cerebrospinal fluid (CSF) biomarkers can be collected via lumbar puncture to provide evidence of amyloid or tau pathology and neurodegeneration. However, lumbar puncture is invasive, requires specialist training and equipment, and is not always acceptable to patients.^10,11^

There has been a drive to develop blood biomarker technology that can provide equivalent information about CSF, and is quick, simple, and cost-effective to deliver. Blood biomarkers could ‘democratize’ the access to Alzheimer’s disease biomarkers, where most UK memory services do not utilize amyloid-PET or CSF technologies.^12,13^ Frisoni *et al*.^14^ set out a strategic roadmap in 2017 to aid the systematic and coordinated research on new biomarker technology with the aim of incorporating them into the clinical setting. They described the clinical validation of Alzheimer’s disease biomarkers through five phases, first by establishing analytical validity, and subsequently clinical validity, and clinical utility, using an approach adapted from the earlier validation of cancer biomarkers. With the advent of disease-modifying treatments for Alzheimer’s disease, there will be increasing onus on the importance of biomarker technology to confirm the presence of amyloid pathology.^15^

The field is investigating other types of biomarkers, specifically focusing on digital biomarkers that integrate artificial intelligence and machine learning.^16,17^ Although still in the developmental stage and less extensively studied compared with blood or CSF biomarkers, these digital biomarkers are now more in demand. It is important to make the distinction between such digital biomarkers, which gather data, and online support tools that aid in synthesizing and contextualizing biomarker information. These digital biomarkers are beyond the scope of this narrative review.

## Challenges in the use of diagnostic technology in clinical practice

The translation of dementia diagnosis biomarker research into clinical practice involves nuance and complexity. While the research sphere offers much information on candidate biomarkers and their potential sensitivity and specificity, there is much less focus on how this technology is integrated into clinical practice. Furthermore, coupled with this potential for greater biomarker access are a number of potential diagnostic challenges for clinicians.^18,19^

First, which diagnostic technology should be used in the clinical assessment? The inherent properties of the investigation may act as barriers to utilization. As mentioned previously, some are physically invasive and costly. There is limited access to specialized tests such as amyloid-PET and MRI in the UK with long waiting times for referral.^8^ In a US simulation study, patients waited, on average, for an estimated 18 months to commence disease-modifying treatments, because of the limitations in diagnostic capacity and the lack of access to amyloid-PET imaging to confirm Alzheimer’s disease pathology.^20^

The patient and carer’s choice is a cornerstone of the diagnostic assessment.^21^ Towards this, the clinician must ascertain how much information they want to receive. Patients will need to be provided with information about the benefits and risks of available biomarker investigations, so that they can make an informed choice.^22^ The clinician will need to feel confident in understanding what these tests can tell and what their limitations are, as well as in conveying this information using understandable and non-jargonistic terms.^23^ It is essential to understand a patient’s wishes and needs when considering whether or not to organize biomarker testing, because patients will differ in how they weigh the pros and cons of testing.^24^ These decisions should occur through a process of shared decision-making, ensuring that it is patient-centred and tailored to each individual.^18^ Yet, in practice, shared decision-making is usually restricted by the information provided.^18,25^

The clinician and patient will also need to consider the number of diagnostic tests appropriate to order and the patient burden associated with it. For example, a patient may be offered a form of neuroimaging alongside a blood biomarker investigation. While this may provide more information on multiple pathological domains, auch as neurodegeneration (CT/MRI scan) and amyloid/tau (blood biomarker), this could introduce direct conflict between two or more test results, or subject patients to unnecessary testing.

How does the clinician interpret the biomarker result? This is a particularly complex task, even in the research space, where blood biomarkers are continuous measures but most of them do not have validated cut-off points for real-world study populations. Ultra-sensitive assays have been developed for the detection of plasma amyloid β pathology, e.g. Aβ42/Aβ40 ratio, and tau pathophysiology, e.g. plasma phosphorylated tau species.^26^ One p-tau181 assay and several plasma Aβ42/Aβ40 ratio assays have received FDA Breakthrough Device Designation in the USA. In Europe, several Aβ42/Aβ40 ratio assays have been CE marked.^27^ Plasma neurofilament light (NfL) chain has been introduced in a UK clinical laboratory with an established reference range.^28^ A clinician may need to manage a test result that is unclear or ambiguous.^29^ Furthermore, currently there is no consensus on the best way to present biomarker results in a way that is meaningful and accessible to both clinicians and patients. The US Alzheimer’s Association recently published the appropriate use of recommendations for blood biomarkers in Alzheimer’s disease, which includes the need to develop tools for the interpretation of results and for communicating them to clinicians and patients.^26^ While the main driver for diagnostic investigation is to increase certainty, this may not always be the outcome after testing.^24^

## New developments in the research field of clinical decision tools and training to support Alzheimer’s disease diagnosis

Computerized clinical decision support systems have been developed to enhance and support clinicians in complex decision-making processes and are categorized by their ability to facilitate support in patient communication, clinical management decisions or diagnostic support, including the interpretation of laboratory results.^30^

While there are many recommendations on the use of such ‘e-tools’ to broadly support clinicians in education and training around biomarker use and communicate this with patients, there is little specific guidance on how this should be achieved.^29^ There are examples of web-based risk-stratifying tools to aid clinician decision-making in other fields of medicine.^31,32^ However, the use of these tools in the diagnosis of Alzheimer’s disease has been less well investigated. Within dementia research, the Alzheimer Biomarkers in Daily Practice project is one of the most comprehensive studies to have addressed this.^19,25^ This group developed a web-based (‘ADappt’) tool for clinicians in the memory clinic, which is designed to calculate the personalized risk estimates of progression from mild cognitive impairment to dementia, to help clinicians interpret biomarker results, communicate results with patients and caregivers and engage patients in shared decision-making regarding diagnostic testing.^19,33^ The pilot data reported that usability was high. Clinicians found the tool was well integrated and easy to use.

No previous systematic review has been conducted to address this area of research, and few published studies addressed this. In this narrative review, we will focus on salient areas in the implementation of this technology to support clinicians alongside recommendations for their further development.

Online tools could be used to support clinicians and patients in a memory clinic setting in the diagnosis of Alzheimer’s disease in the following ways:

1. To help clinicians decide which biomarker investigation (and any further tests) to order through shared decision-making with patients.
2. To support the interpretation of biomarker test results to an individual.
3. To calculate an individual’s risk score.
4. To support clinicians in explaining and communicating biomarker test results.

A summary table of the available online tools is reported in Table 1.

**Table 1:** A summary table of available online tools to support clinicians and patients in a memory clinic setting

	Tool	Summary of characteristics	Strengths	Limitations
Information on biomarker quality	ADappt tool	Provides a summary of currently available diagnostic tests in the memory clinic. Each test is summarized with information provided on test purpose, pros and cons	Interactive and easy to understand summaries. High usability. Effective design and interface	Difficulty in maintaining up-to-date information
Decision support aids	ADappt tool	Provides guidance on how to structure a discussion around diagnostic testing to facilitate shared decision-making	Example phrases are constructed	The tool is not integrated within electronic medical records
Biomarker test interpretation	ADappt tool	Summary of the information available from each diagnostic test, followed by the interpretation of the test result. Explanation on how the test result affects the chance of Alzheimer’s disease dementia	Simplified summary of MRI head results, e.g. definite atrophy/possible atrophy/no atrophy	Possibility of conflicting test results
	Plasma neurofilament light chain interface for physicians	Online tool. The clinician enters the patient’s age and plasma neurofilament light chain result. The result is plotted on a figure that shows the result in the context of the percentile neurofilament light chain levels of controls and other diagnoses	Personalized and interactive tool. The summary information provided on neurofilament light chain and its interpretation	Data are not currently available to interpret the neurofilament light chain level in the context of other co-morbidities, e.g. renal function or body mass index
Risk score calculators	ADappt tool	Risk calculation module. Risk score calculator to understand an individual’s risk of progression from mild cognitive impairment to Alzheimer’s disease dementia based on patient demographics and biomarker results	The risk of 1- and 3-year progression from mild cognitive impairment to Alzheimer’s disease dementia is provided as a percentage score	Clinicians must be appropriately trained to provide information on risk to patients
	Cates Plot	A visual decision aid, usually to explain the number needed to treat when comparing placebo with treatment. Available for commencing/stopping a cholinesterase inhibitor and antipsychotic medications in dementia	Effective visualization strategy. This tool can be used to support shared decision-making with patients	Requires adequate explanation by the clinician to ensure patients understand the data presented
Support in communicating results	ADappt tool	Provides a personalized printable report sheet with a text explanation and their investigation results	A clear and concise overview of information	Currently unable to provide patient’s personalized MRI brain image, instead uses a stock image

## Deciding which biomarker investigation to order through shared decision-making with patients

Clinicians need access to up-to-date information on the qualities of individual biomarkers and their potential benefits and drawbacks.^34^ This information can then be distilled and discussed with patients. A clinician will often search for available information from several sources.^35^ An online tool could provide a pooled resource summary of available evidence and information. The ADappt tool contains summarized information on current diagnostic tests available in the memory clinic, as well as the test’s purpose, strengths and limitations.^33,36^

Shared decision-making is an approach within which clinicians and patients, together with caregivers, share evidence and understand and agree with individualized preferences to reach a decision when there is more than one option available.^37^ Shared decision-making is appropriate in the diagnostic assessment of Alzheimer’s disease where more than one diagnostic option may be available.^19^ Shared decision-making in the setting of cognitive impairment may be more complex and will often require a collaborative approach involving the patient, clinician and another individual, such as the next of kin, an individual with Lasting Power of Attorney for health or someone in a caregiving role. Decision aids can assist in this shared decision-making. However, few are available in public access websites.^38^ Further work is required to use thee consistent plain language terminology for biomarkers when communicating with patients and their families.^23^ Accessing online-constructed example phrases may provide a conversation starter for clinicians to involve patients in shared decision-making.^19^

## To support the interpretation of biomarker test results to an individual

Currently, biomarkers in the clinical setting (e.g. blood biomarkers) do not have validated health disease cut-points.^26^ Clinicians who are used to having biomarker reference ranges for health and disease would need to interpret this result. Consideration is needed on how to present the result data and provide context for interpretation. This could involve calculating potential confounding factors or adjustments that may be necessary, such as weight or renal function. In this scenario, an online tool could serve as a prompt for the clinician to actively consider these factors and perform the required calculations and adjustments.^30,39^ This result could be plotted on a graph, e.g. using biomarker data ranges available from validation cohorts.^40^ The NfL interface for physicians is an online tool in the research setting to aid clinicians in interpreting the plasma NfL levels in The Netherlands.^41^ The clinician enters the patient’s age and plasma NfL result. The result is plotted on a figure, which shows the result in the context of the percentile NfL levels of controls and other diagnoses.

**Figure 1:**
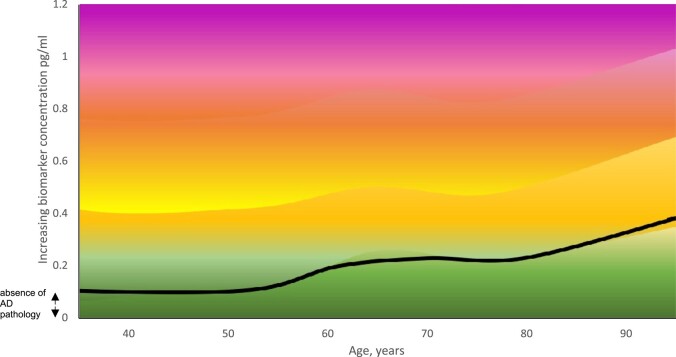
**Estimating the risk of Alzheimer’s disease with increasing concentration of a hypothetical Alzheimer’s disease biomarker by increasing age (years).** The black line represents the threshold between the presence or absence of Alzheimer’s disease pathology. Alzheimer’s disease pathology risk thresholds are represented by a graduation from green (low risk) to red (high risk).

Computerized diagnostic systems have been programmed to perform automated tumour grading, electrocardiographic analysis, and arterial blood gas interpretation.^30^ Unidimensional results are relatively easy for clinicians to interpret and have clearly defined decision boundaries. However, the transition from a normal to an abnormal result is more gradual with an Alzheimer’s disease blood biomarker. The clinician may also need to hold in mind several parameters that can affect the test result interpretation. The parameters may include several biomarker results that need to be combined, such as a patient’s age and another patient-specific factor, e.g. renal function. It therefore becomes increasingly difficult to ‘visualize’ the separation between a normal and an abnormal result.^42^ This is illustrated using a hypothetical Alzheimer’s disease biomarker in Fig. 1. This figure illustrates just two parameters that need to be considered: age and biomarker result.

A pilot study to assess the acceptability of a blood biomarker, plasma phosphorylated–tau181 in a UK memory service, reported that clinicians wanted access to additional online tools to aid in the interpretation of the result.^40^ This included video information and an interactive website with educational elements. Simulated case vignettes with multiple-choice biomarker results could provide clinicians with active examples of biomarker interpretation and mimic the clinical scenarios they may encounter.^43^ An e-tool could also guide the clinician in the need for further investigations.

## To calculate an individual’s risk score

Clinician decision support tools can help clinicians in managing the amount and array of available information in the diagnostic assessment of Alzheimer’s disease. The healthcare setting is sometimes seen as ‘information rich’ but ‘knowledge poor’.^44,45^ While within healthcare systems there is an abundance of available data, analysis methods are required to extract pertinent information. Analysis tools can aid clinicians in making sense of and extracting meaningful information from the data. Some tools can be designed by drawing on large datasets, using algorithms to detect patterns, and supporting clinician decision-making using risk stratification, through novel machine learning.^45^

Online risk score calculators are available to support clinicians in their decision-making in several medical specialties and have been successfully integrated into clinical practice. The Fracture Risk Assessment Tool is a frequently used fracture risk calculator.^46^ Adjuvant Online is a tool used in oncology to calculate the risk of breast cancer recurrence if patients are treated with or without adjuvant therapy.^19,32^ The Q-RISK score and U-Prevent are online tools to calculate therapy benefits related with cardiovascular risk prevention.^31,47^

Decision aids are available to support clinicians in prescribing antidementia medications (benefit) and antipsychotics (risk of harm) in the UK.^48,49^ An individual’s risk score is sometimes depicted using a Cates plot,^50^ which is a visual decision aid that provides four face categories, via a traffic light system, to explain the risk or benefit of an event (usually the number needed to treat) when comparing placebo with treatment in 100 people.^50-52^ These tools and any future online calculator for Alzheimer’s disease biomarker results could also provide further education and training for clinicians.

A risk score calculator could assist a clinician in a memory clinic setting to understand an individual’s risk of progression from mild cognitive impairment to Alzheimer’s disease dementia, as illustrated in the ‘ADappt’ tool or alternatively via a composite score to assess an individual’s probability of Alzheimer’s disease by drawing information on demographic and biomarker data.^19,53^ As the number of biomarkers increases, there will be a growing need for tools that can address differential diagnosis in a more nuanced manner. Currently, the most established markers identify the presence or absence of Alzheimer’s disease amyloid pathology. However, with advancements in research, it is anticipated that a wider array of biomarkers may become available in the future, providing information for differential diagnoses including Alzheimer’s disease, neurodegeneration or Lewy body pathology, among others.

## Support clinicians in explaining and communicating biomarker test results

There are no current guidelines for communicating Alzheimer’s disease biomarker results in a clinic setting.^54^ There is, however, detailed guidance published in research settings for explaining amyloid-PET results to individuals who are asymptomatic or have a diagnosis of mild cognitive impairment.^55-57^ These communication models use standardized language when describing the clinical significance of amyloid biomarkers.^58^ Adjuncts to support clinicians in explaining results could be via interactive visual aids such as a traffic light system, where green indicates normal, amber intermediate and red abnormal biomarker results.^19^

Patients could be provided with a personalized printable report sheet with text explanation and their investigation results.^19^ This could include a stock image of, for example, an MRI head result or the patient’s personalized image.

## Barriers to implementation

There are barriers in the implementation of an e-tool in the clinical pathway and its translation from the research setting, and there may be difficulty in integrating tools into the existing workflow.^59,60^ Clinicians need to be aware of existing technology and buy into its use. Any information or data will need to be kept up to date and in line with a developing field. Separation of any e-tool from existing electronic medical records could create extra work for the clinician and a lack of harmony if it does not marry up with the existing system. An e-tool that uses existing system data must comply with data security standards such as General Data Protection Regulation.^30^ The cost of implementing, integrating and maintaining an e-tool must be justified.^30^ Clinicians have expressed a distrust of the output from such tools in previous studies.^61^ When piloting the ‘ADappt’ tool in Dutch memory services, barriers faced included technical difficulty, being blocked by the trust firewall and clinicians not having time available to use an additional e-tool in a consultation.^19^ Clinicians overcame these barriers by using the tool on a different device and using a discrete section of the website, e.g. the results page in the clinical discussion.

## Conclusion and recommendations for future directions

Currently, there have been few studies that explore the use of online tools to support clinicians in using new diagnostic technology for Alzheimer’s disease. Predominantly, the published literature has explored the use of an e-tool to support clinicians and patients in communicating biomarker results and the risk of progression in mild cognitive impairment. Further work is needed to develop effective e-tools, which must be both acceptable to clinicians, patients and carers and easily integrated into the local or national clinical pathway to ensure the feasibility of use. Such e-tools should be designed to provide support in key areas of Alzheimer’s disease diagnosis: ordering biomarker investigations, calculating individual risk scores and interpretation and communication of results.

## Data Availability

Data sharing is not applicable to this article as no new data were created or analysed in this study.

## References

[1] Jack CR Jr , Bennett DA, Blennow K, et al NIA-AA research framework: Toward a biological definition of Alzheimer’s disease. Alzheimers Dement. 2018;14(4):535–562. 29653606 10.1016/j.jalz.2018.02.018 PMC5958625

[2] Chapleau M , Iaccarino L, Soleimani-Meigooni D, Rabinovici GD. The role of amyloid PET in imaging neurodegenerative disorders: A review. J Nucl Med. 2022;63(Supplement 1):13S–19S. 35649652 10.2967/jnumed.121.263195 PMC9165727

[3] Dubois B , Villain N, Frisoni GB, et al Clinical diagnosis of Alzheimer’s disease: Recommendations of the international working group. Lancet Neurol. 2021;20(6):484–496. 33933186 10.1016/S1474-4422(21)00066-1 PMC8339877

[4] The National Institute on Aging and the Alzheimer’s Association . NIA-AA revised diagnostic criteria: A biological definition of Alzheimer’s disease. Accessed 29 September 2023. https://aaic.alz.org/nia-aa.asp#workgroup

[5] Jack CRJ , Petersen RC, Xu YC, et al Medial temporal atrophy on MRI in normal aging and very mild Alzheimer’s disease. Neurology. 1997;49(3):786–794. 9305341 10.1212/wnl.49.3.786 PMC2730601

[6] Barthel H , Sabri O. Clinical use and utility of amyloid imaging. J Nucl Med. 2017;58(11):1711–1717. 28818990 10.2967/jnumed.116.185017

[7] Suppiah S , Didier MA, Vinjamuri S. The who, when, why, and how of PET amyloid imaging in management of Alzheimer’s disease—Review of literature and interesting images. Diagnostics. 2019;9(2):65. 31242587 10.3390/diagnostics9020065 PMC6627350

[8] Royal College of Psychiatrists DAT, Alzheimer’s Research UK . Are we ready to deliver disease modifying treatments? 2021. Accessed 18 April 2023. https://www.alzheimersresearchuk.org/wp-content/uploads/2021/05/ARUK-Are-we-ready-to-deliver-disease-modifying-treatments_25May21.pdf

[9] Rabinovici GD , Chaudhary K, Lesman-Segev O, et al Impact of amyloid PET on the management of cognitively impaired patients: Results from the ideas study. J Prev Alzheimers Dis. 2018;5(1):S13–S14.

[10] Duits FH , Martinez-Lage P, Paquet C, et al Performance and complications of lumbar puncture in memory clinics: Results of the multicenter lumbar puncture feasibility study. Alzheimers Dement. 2016;12(2):154–163. 26368321 10.1016/j.jalz.2015.08.003

[11] Hampel H , Vergallo A, Shaw LM, et al State-of-the-art of lumbar puncture and its place in the journey of patients with Alzheimer’s disease. Alzheimers Dement. 2022;18(1):159–177. 34043269 10.1002/alz.12372 PMC8626532

[12] Cook LD , Nichol KE, Isaacs JD. The London memory service audit and quality improvement programme. BJPsych Bull. 2019;43(5):215–220. 10.1192/bjb.2019.18 PMC12402916 30898180

[13] O’Brien JT , Herholz K. Amyloid imaging for dementia in clinical practice. BMC Med. 2015;13(1):1–3. 26170121 10.1186/s12916-015-0404-6 PMC4499896

[14] Frisoni GB , Boccardi M, Barkhof F, et al Strategic roadmap for an early diagnosis of Alzheimer’s disease based on biomarkers. Lancet Neurol. 2017;16(8):661–676. 28721928 10.1016/S1474-4422(17)30159-X

[15] Sims JR , Zimmer JA, Evans CD, et al Donanemab in early symptomatic Alzheimer disease: The TRAILBLAZER-ALZ 2 randomized clinical trial. JAMA. 2023;330(6):512–527. 37459141 10.1001/jama.2023.13239 PMC10352931

[16] Kourtis LC , Regele OB, Wright JM, Jones GB. Digital biomarkers for Alzheimer’s disease: The mobile/wearable devices opportunity. NPJ Digit Med. 2019;2(1):9. 31119198 10.1038/s41746-019-0084-2 PMC6526279

[17] Dorsey ER , Papapetropoulos S, Xiong M, Kieburtz K. The first frontier: Digital biomarkers for neurodegenerative disorders. Digit Biomark. 2017;1(1):6–13. 32095743 10.1159/000477383 PMC7015357

[18] Kunneman M , Smets EM, Bouwman FH, et al Clinicians’ views on conversations and shared decision making in diagnostic testing for Alzheimer’s disease: The ABIDE project. Alzheimers Dement. 2017;3(3):305–313. 10.1016/j.trci.2017.03.009 PMC5651435 29067337

[19] van Buchem M , Zwan MD, Pelkmans W, et al Development and usability of ADappt: An online tool to support clinicians, patients and caregivers in the diagnosis of mild cognitive impairment and Alzheimer’s disease. Alzheimers Dement. 2019;15(7 Supplement):P163–P164. 10.2196/13417 PMC6643768 31287061

[20] Liu J , Hlávka J, Hillestad RJ, Mattke S. Assessing the preparedness of the US health care system infrastructure for an Alzheimer’s treatment. RAND Corporation; 2017. 10.7249/rhq-08-03-02 PMC6557037 31205802

[21] Pink J , O’Brien J, Robinson L, Longson D. Dementia: Assessment, management and support: Summary of updated NICE guidance. BMJ. 2018;361:k2438. 29925626 10.1136/bmj.k2438

[22] Tariot P . Blood-based biomarkers for Alzheimer’s disease: Are we there yet? J Prev Alzheimers Dis. 2022;9(4):565–566. 36281659 10.14283/jpad.2022.88

[23] Martin N , Dropkin L, Molina M, Redway L. P43.02 inconsistencies within biomarker test reports provide opportunities for future patient education. J Thorac Oncol. 2021;16(10):S1082–S1083.

[24] Kunneman M , Pel-Littel R, Bouwman FH, et al Patients’ and caregivers’ views on conversations and shared decision making in diagnostic testing for Alzheimer’s disease: The ABIDE project. Alzheimers Dement. 2017;3(3):314–322. 10.1016/j.trci.2017.04.002 PMC5651429 29067338

[25] de Wilde A , van Maurik IS, Kunneman M, et al Alzheimer’s biomarkers in daily practice (ABIDE) project: Rationale and design. Alzheimers Dement. 2017;6:143–151. 10.1016/j.dadm.2017.01.003 PMC5318541 28239639

[26] Hansson O , Batrla R, Brix B, et al The Alzheimer’s Association international guidelines for handling of cerebrospinal fluid for routine clinical measurements of amyloid beta and tau. Alzheimers Dement. 2021;17(9):1575–1582. 33788410 10.1002/alz.12316

[27] Iaccarino L , Burnham S, Dell’Agnello G, Dowsett S, Epelbaum S. Diagnostic biomarkers of amyloid and tau pathology in Alzheimer’s disease: An overview of tests for clinical practice in the United States and Europe. J Prev Alzheimers Dis. 2023;10(3):426–442. 37357283 10.14283/jpad.2023.43

[28] Simrén J , Andreasson U, Gobom J, et al Establishment of reference values for plasma neurofilament light based on healthy individuals aged 5–90 years. Brain Commun. 2022;4(4):fcac174. 35865350 10.1093/braincomms/fcac174 PMC9297091

[29] van Maurik IS , Zwan MD, Tijms BM, et al Interpreting biomarker results in individual patients with mild cognitive impairment in the Alzheimer’s Biomarkers in Daily Practice (ABIDE) project. JAMA Neurol. 2017;74(12):1481–1491. 29049480 10.1001/jamaneurol.2017.2712 PMC5822193

[30] Sutton RT , Pincock D, Baumgart DC, Sadowski DC, Fedorak RN, Kroeker KI. An overview of clinical decision support systems: Benefits, risks, and strategies for success. NPJ Digit Med. 2020;3(1):17. 32047862 10.1038/s41746-020-0221-y PMC7005290

[31] Jaspers NE , Ridker PM, Dorresteijn JA, Visseren FL. The prediction of therapy-benefit for individual cardiovascular disease prevention: Rationale, implications, and implementation. Curr Opin Lipidol. 2018;29(6):436–444. 30234556 10.1097/MOL.0000000000000554

[32] Paridaens RJ , Gelber S, Cole BF, et al Adjuvant! © Online estimation of chemotherapy effectiveness when added to ovarian function suppression plus tamoxifen for premenopausal women with estrogen-receptor-positive breast cancer. Breast Cancer Res Treat. 2010;123:303–310. 20195744 10.1007/s10549-010-0794-2 PMC3588884

[33] van Maurik IS , Visser LN, Pel-Littel RE, et al Development and usability of ADappt: Web-based tool to support clinicians, patients, and caregivers in the diagnosis of mild cognitive impairment and Alzheimer disease. JMIR Form Res. 2019;3(3):e13417. 31287061 10.2196/13417 PMC6643768

[34] Hampel H , Au R, Mattke S, et al Designing the next-generation clinical care pathway for Alzheimer’s disease. Nature Aging. 2022;2(8):692–703. 37118137 10.1038/s43587-022-00269-x PMC10148953

[35] Davies K . The information-seeking behaviour of doctors: A review of the evidence. Health Info Libr J. 2007;24(2):78–94. 17584211 10.1111/j.1471-1842.2007.00713.x

[36] ADappt . Accessed 29 September 2023. https://www.adappt.health/nl/informatie

[37] Elwyn G , Frosch D, Thomson R, et al Shared decision making: A model for clinical practice. J Gen Intern Med. 2012;27:1361–1367. 22618581 10.1007/s11606-012-2077-6 PMC3445676

[38] Coulter A , Edwards A, Elwyn G, Thomson R. Implementing shared decision making in the UK. Z Evid Fortbild Qual Gesundheitswes. 2011;105(4):300–304. 10.1016/j.zefq.2011.04.014 21620325

[39] Mørkrid L , Rowe AD, Elgstoen KB, et al Continuous age-and sex-adjusted reference intervals of urinary markers for cerebral creatine deficiency syndromes: A novel approach to the definition of reference intervals. Clin Chem. 2015;61(5):760–768. 25759465 10.1373/clinchem.2014.235564

[40] Hazan J , Hall S, Pemberton A, et al Acceptability and feasibility of plasma phosphorylated-tau181 in two memory services. Int J Geriatr Psychiatry. 2023;38(3):e5897. 36852663 10.1002/gps.5897

[41] NfL interface for physicians . Published 9 March 2023. Accessed 29 September 2023. https://mybiomarkers.shinyapps.io/Neurofilament/

[42] Pisner DA , Schnyer DM. Support vector machine. In: Machine learning. Elsevier; 2020:101–121.

[43] Gooblar J , Carpenter BD, Coats MA, Snider BJ, Morris JC. The influence of cerebrospinal fluid (CSF) biomarkers on clinical dementia evaluations. Alzheimers Dement. 2015;11(5):533–540. 25022536 10.1016/j.jalz.2014.04.517 PMC4287458

[44] AbuKhousa E , Campbell P. Predictive data mining to support clinical decisions: An overview of heart disease prediction systems. IEEE; 2012:267–272.

[45] Bellio M , Furniss D, Oxtoby NP, et al Opportunities and barriers for adoption of a decision-support tool for Alzheimer’s disease. ACM Trans Comput Healthc. 2021;2(4):1–19.

[46] Lekamwasam S . The diversity of fracture risk assessment tool (FRAX)-based intervention thresholds in Asia. Osteoporosis Sarcopenia. 2019;5(4):104–108. 31938728 10.1016/j.afos.2019.12.002 PMC6953527

[47] Hippisley-Cox J , Coupland C, Vinogradova Y, Robson J, May M, Brindle P. Derivation and validation of QRISK, a new cardiovascular disease risk score for the United Kingdom: Prospective open cohort study. BMJ. 2007;335(7611):136. 17615182 10.1136/bmj.39261.471806.55 PMC1925200

[48] Carmody J , Traynor V, Steele A. Dementia, decision aids and general practice. Australian Family Physician. 2015;44(5):307–310. 26042403

[49] National Institute for Healthcare and Excellence . Antipsychotic medicines for treating agitation, aggression and distress in people living with dementia .2018 Accessed 01 December 2023. https://www.nice.org.uk/guidance/ng97/resources/antipsychotic-medicines-for-treating-agitation-aggression-and-distress-in-people-living-with-dementia-patient-decision-aid-pdf-4852697005.

[50] Liou YL , Urban K. 35302 assessment of the impact of Cates plot on patient comprehension of patch test predictive value. J Am Acad of Dermatol. 2022;87(3):AB19..

[51] Dr Chris Cates . Cates plot. Accessed 20 March 2023. https://www.nntonline.net/visualrx/cates_plot/

[52] Venekamp RP , Sanders SL, Glasziou PP, Del Mar CB, Rovers MM. Antibiotics for acute otitis media in children. Cochrane Database Syst Rev. 2015;2015(6):CD000219. 26099233 10.1002/14651858.CD000219.pub4 PMC7043305

[53] Reitz C , Tang MX, Schupf N, Manly JJ, Mayeux R, Luchsinger JA. A summary risk score for the prediction of Alzheimer disease in elderly persons. Arch Neurol. 2010;67(7):835–841. 20625090 10.1001/archneurol.2010.136 PMC3068839

[54] Milne R , Bunnik E, Diaz A, et al Perspectives on communicating biomarker-based assessments of Alzheimer’s disease to cognitively healthy individuals. J Alzheimers Dis. 2018;62(2):487–498. 29480179 10.3233/JAD-170813 PMC5836405

[55] Burns JM , Johnson DK, Liebmann EP, Bothwell RJ, Morris JK, Vidoni ED. Safety of disclosing amyloid status in cognitively normal older adults. Alzheimers Dement. 2017;13(9):1024–1030. 28263740 10.1016/j.jalz.2017.01.022 PMC5582024

[56] Harkins K , Sankar P, Sperling R, et al Development of a process to disclose amyloid imaging results to cognitively normal older adult research participants. Alzheimers Res Ther. 2015;7(1):1–9. 25969699 10.1186/s13195-015-0112-7 PMC4428104

[57] Lingler JH , Butters MA, Gentry AL, et al Development of a standardized approach to disclosing amyloid imaging research results in mild cognitive impairment. J Alzheimers Dis. 2016;52(1):17–24. 27060950 10.3233/JAD-150985 PMC4860948

[58] Visser PJ , Wolf H, Frisoni G, Gertz HJ. Disclosure of Alzheimer’s disease biomarker status in subjects with mild cognitive impairment. Biomark Med. 2012;6(4):365–368. 22917137 10.2217/bmm.12.58

[59] Musen MA , Middleton B, Greenes RA. Clinical decision-support systems. In: Biomedical informatics: Computer applications in health care and biomedicine. Springer; 2021:795–840.

[60] Wears RL , Berg M. Computer technology and clinical work: Still waiting for Go. JAMA. 2005;293(10):1261–1263. 15755949 10.1001/jama.293.10.1261

[61] Yang Q , Steinfeld A, Zimmerman J. Unremarkable AI: Fitting intelligent decision support into critical, clinical decision-making processes. In CHI Conference on Human Factors in Computing Systems Proceedings (CHI 2019), May 4–9, 2019, Glasgow, Scotland, UK. ACM. 2019:1–11.

